# Enzymatic biofilm destabilisation to support mechanical cleansing of inserted dental implant surfaces: an in-vitro pilot study

**DOI:** 10.1007/s10266-021-00599-z

**Published:** 2021-03-19

**Authors:** Rutger Matthes, Lukasz Jablonowski, Birte Holtfreter, Christiane Pink, Thomas Kocher

**Affiliations:** Department of Restorative Dentistry, Periodontology, Endodontology, Preventive and Pediatric Dentistry, Dental School, University Medicine, Greifswald Rotgerberstr. 8, 17475 Greifswald, Germany

**Keywords:** Cocamidopropyl betaine, Dental biofilm, EDTA, Enzymes, Implant, Titanium

## Abstract

Peri-implantitis is caused by microbial contamination and biofilm formation on the implant surface. To achieve re-osseointegration, the microbes must be completely removed from the surface. Adjunctive to mechanical cleaning, chemical treatment with enzymes or other substances could optimise the treatment outcome. Therefore, we investigated the efficacy of different enzymes, a surfactant, and a chelator in destabilising dental polymicrobial biofilm. The biofilm destabilising effect of the glycosidases α-amylase, dextranase, DispersinB^®^, and lysozyme, as well as the proteinase subtilisin A, and the nuclease Benzonase^®^, the chelator EDTA, and the surfactant cocamidopropyl betaine were investigated on biofilms, inoculated with plaque on rough titanium discs. The test and the control solutions were incubated for 15 min at 36 °C on biofilms, and loosened biofilm mass was removed by shear stress with a shaker. Fluorescence-stained biofilms were microscopically analysed. Acceptable cell tolerability concentrations of test substances were determined by the MTT (tetrazolium dye) assay on the MG-63 cell line. A statistically significant biofilm destabilising effect of 10% was shown with lysozyme (2500 µg/ml).

## Introduction

Currently, approximately 12 million implants per year are placed worldwide [1], with the number inserted dental implants increasing continuously. Correspondingly, the number of peri-implantitis cases is expected to rise [2]. About 28% of all implants develop peri-implantitis with bone loss [3]. A Swedish study calculated that 4.2% of implant wearers lost their implant within 9 years, which amounted to 2% of all inserted implants [4].

Peri-implantitis can have various causes [5]. At present, most researchers consider microbial contamination and the development of plaque biofilm along the implant as the cause of peri-implantitis with its ensuing inflammation and bone loss.

Microorganisms of various species are organised in a consortium and are embedded in a hydrated matrix of polysaccharides and polypeptides, which is synthesised by the microorganisms themselves and accumulates from the environment. The biofilm matrix promotes microbial tolerance against the host defence system or antimicrobial and antibiotic treatment [6, 7]. The microrough surface of implants supports bacterial adherence and provides niches for microorganisms, which increase the risk that microorganisms will settle and survive on the implant surface and impede surface cleansing in the case of peri-implantitis therapy.

To achieve re-osseointegration, in addition to a surgical approach, such as guided bone regeneration, the microbes must be completely removed from the implant surface. Complete cleaning is challenging not only because of the complex macrogeometry of the implant body but also because of the rough microstructure of the implant surface, which both hampers instrument access and impedes biofilm removal. Residual microbes can reorganise into a biofilm and revive the inflammation process. This background shows that thorough debridement is important. A strategy to destabilise the biofilm matrix could improve the efficacy of mechanical implant cleaning during a flap surgery by removing microorganisms from implant areas that are difficult to access and treat. If chemical substances are used during surgery, they must not interfere with wound healing by damaging cells.

The stability and surface adherence of the biofilm matrix is mainly affected by polysaccharides, polypeptides, and often by extracellular DNA. Negative charges of chemical functional groups embed cations, e.g., Ca^2+^ and Mg^2+^, which further stabilise the biofilm matrix [8, 9].

There are multiple targets for destabilising the microbial consortium. For instance, the microbial cell membranes and cell walls contain lipids, glycolipids and integrated membrane proteins linked with extracellular polysaccharides. Enzymes which can break glycoside or peptide bonding, or a surfactant, which disturbs polar interactions, or chelators which bind cations, could be helpful tools to destabilise biofilm matrix.

The aim of this pilot study was to evaluate the ability of different enzymes, one chelator and one surfactant to destabilise dental plaque biofilm after identifying tissue-tolerable concentrations for each substance. This study is to be considered as a baseline study; future studies will investigate the effectiveness of more complex enzyme mixtures and other additives to support mechanical cleaning. We hypothesised that one or more test substances, used in tissue-tolerable concentrations, would destabilise the biofilm matrix of the experimental dental in-vitro biofilm and that subsequent mechanical shear stress (shaking) would significantly reduce the biofilm mass in comparison to the control.

## Materials and methods

**Fig. 1:**
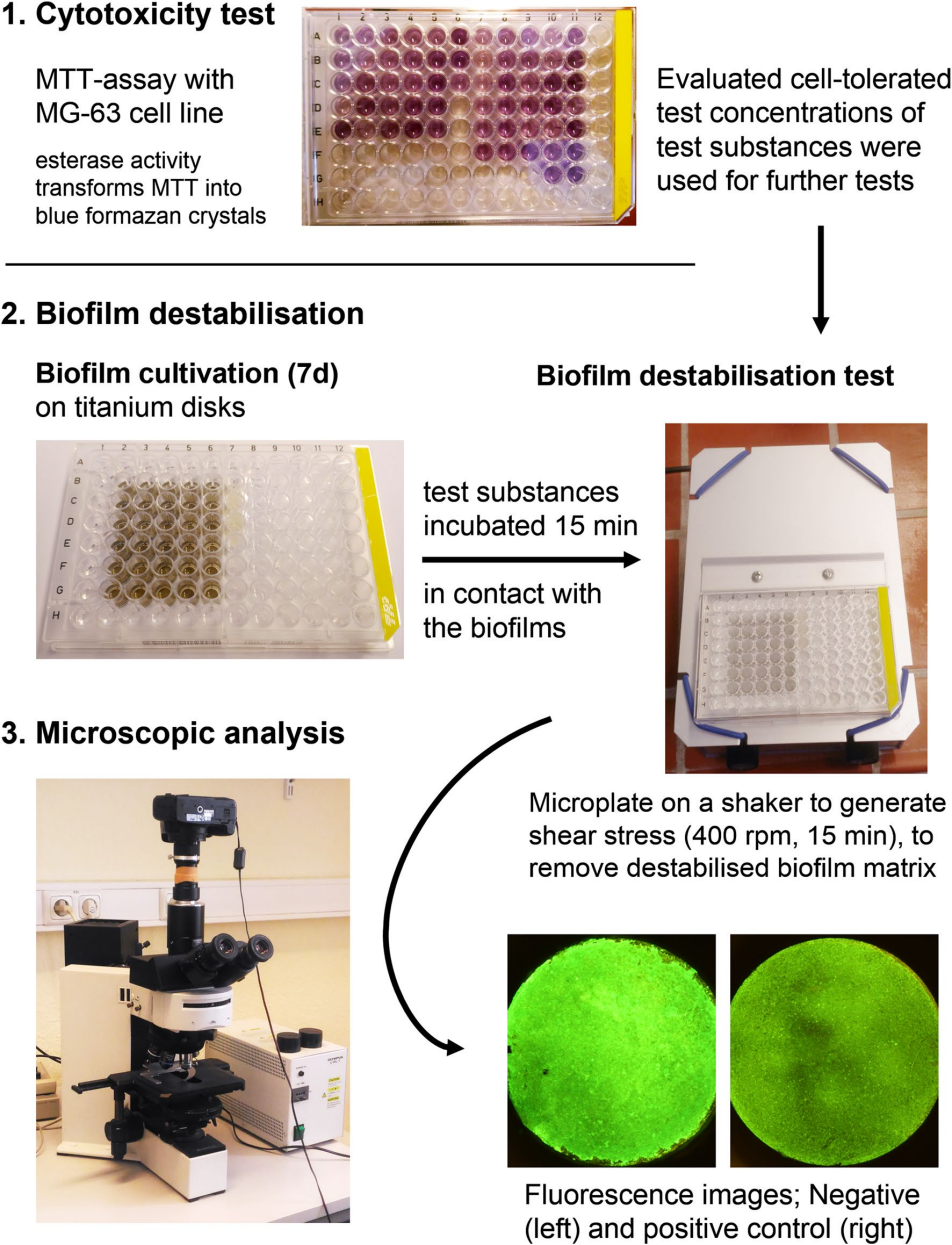
Scheme of the test process. The cell-tolerated concentrations of the test substances were evaluated and tested on 7-day-old plaque biofilm. Potential biofilm destabilising effects were identified by fluorescence microscopic analysis after shaking treated specimens

Besides activity tests of the enzymes used, the limits of cell tolerability of the test substances were determined prior to starting the tests. These concentrations were used to investigate their biofilm destabilising effect. The sequence of the test procedure is shown in Fig. 1.

### Test substances and activity tests

The following enzymes were tested:

α-Amylase from *Bacillus subtilis* (Sigma-Aldrich, Munich, Germany), a glycoside hydrolase which acts on α-1,4-glycosidic bonds (EC 3.2.1.1), was used with 6000 µg/ml in Dulbecco's Phosphate-Buffered Saline (DPBS, Carl Roth, Karlsruhe, Germany). The activity, expressed in U/mg (units/mg) solid, was tested using the PHADEBAS Amylase test (magle Life Sciences, Lund, Sweden).

Benzonase^®^ (Merck-Millipore, Darmstadt, Germany), an endonuclease (EC 3.1.30.2), was used with 0.07 µg/ml in DPBS with added magnesium chloride hexahydrate (SERVA, Heidelberg, Germany) (final concentration 1 mM Mg^2+^). The activity, expressed in U/mg protein, was tested according to the protocol of the Benzonase^®^ Brochure (2013) – document 200411.164 of Merck Millipore.

Dextranase from *Penicillium sp*. (Sigma-Aldrich, Munich, Germany), a glucanohydrolase which acts on (1->6)-α-d-glucosidic linkages in dextran (EC 3.2.1.11), was used at a concentration of 200 µg/ml in DPBS. The activity, expressed in U/mg solid, was tested according to the protocol of Janson and Porath [10, 11].

DispersinB^®^ B from *Actinobacillus actinomycetemcomitans* (BioVectra, Charlottetown, PE, Canada), a hexosaminidase which acts on *N*-acetyl-d-hexosamine residues (EC 3.2.1.52), was used at a concentration of 250 µg/ml in DPBS. The activity, expressed in U/mg protein, was tested according to the protocol of Shibata and Yagi [12] using 4-nitrophenyl *N*-acetyl-β-d-glucosaminide as the substrate.

Lysozyme from hen’s egg protein (Carl Roth, Karlsruhe, Germany), a muramidase which acts on (1->4)-β-linkages between *N*-acetylmuramic acid and *N*-acetyl-d-glucosamine residues (EC 3.2.1.17), was used at concentrations of 2500 and 5000 µg/ml in DPBS. The activity, expressed in U/mg solid, was tested according to the protocol of Shugar [13, 14] by means of *Micrococcus lysodeikticus* (ATCC No. 4698) lyses. The extinction was measured at 450 nm (TriStar LB941, Berthold Technologies, Bad Wildbad, Germany).

Subtilisin A from *Bacillus licheniformis* (Sigma-Aldrich, Munich, Germany), a serine endopeptidase which acts on peptide bonds (EC 3.4.21.62), was used at a concentration of 2 µg/ml in DPBS. The activity, expressed in U/mg solid, was tested following the Sigma Quality Control Test Procedure—Enzymatic Assay of Protease [15] with casein as the substrate and Folin and Ciocalteu’s Phenol reagent for analysis.

The absorptions of the different activity assays were measured using a spectrophotometer (PowerWave XS, BioTek Instruments, Bad Friedrichshall, Germany).

In addition to the enzymes, the following chemical substances were tested:

Cocamidopropyl betaine (CAPB, Spinnrad, Bad Segeberg, Germany), a surfactant (CAS № 61789-40-0), was used at a concentration of 10 µg/ml in DPBS without Mg^2+^ und Ca^2+^ (Carl Roth, Karlsruhe, Germany). CAPB was additionally used as a positive control at 2 or 5% (20 or 50 mg/ml).

Ethylenediaminetetraacetic acid (EDTA, VWR Chemicals, Darmstadt, Germany), a chelating agent (CAS № 60-00-4), was used at a concentration of 292 µg/ml (0.001 M) in DPBS without Mg^2+^ und Ca^2+^.

### Cytotoxicity test

To evaluate the cytotoxicity of enzymatic solutions in different concentrations, human osteoblastic cells (MG-63; RRID:CVCL_0426, ATCC, CRL-1427; LGC Promochem, Wesel, Germany) were cultured in DMEM with 10% FCS in T75-cell culture flasks (TPP, Trasadingen, Switzerland) at 37 °C in a humidified atmosphere with 5% CO_2_, and were split at 80% confluence to obtain an adequate number of cells for the cytotoxicity test. Cells were seeded in a 96-well microplate (TPP, Trasadingen, Switzerland) with 100 µl of 2 × 10^4^ cells/ well. After 24-h incubation, the cytotoxicity test using the MTT assay (3-(4,5-dimethylthiazol-2-yl)-2,5-diphenyltetrazolium bromide), which is based on cell esterase activity, was performed. Therefore, the medium was removed and 70 µl of pre-tempered test substances were transferred into respective test wells. After 15 min of incubation at 36 °C/5% CO_2_, the test solutions were removed, and each well was washed once with 150 µl DPBS. Subsequently, wells with 100 µl pre-tempered MTT culture medium (0.5 mg MTT/ml DMEM + 10%FBS) were incubated for 4 h at 37 °C/5% CO_2_. In order to determine the reduction product formed (formazan crystals), the MTT medium was removed, the wells were washed once with PBS, and 200 µl elution medium (2-Propanol with 4% 1 M HCl, both from Carl Roth, Karlsruhe, Germany) was used to elute the formazan. For analysis, the elution was measured spectrophotometrically at 540 nm (reference at 655 nm) with the microplate reader PowerWave XS (Biotek Instruments, Germany).

The cytotoxicity tests served as the basis for finding an acceptable application concentration in biofilm destabilisation tests. The average reduced viability should not drop below 50% compared to the DPBS control. The 50% limit was chosen following guidelines for the similar XTT assay (2,3-Bis(2-methoxy-4-nitro-5-sulfophenyl)-5-((phenylamino)carbonyl)-2H-tetrazoliumhydroxid assay), as stated in DIN EN ISO 10993-5:2007 [16]. The cytotoxicity tests were run 1 or more times (3 times in average) for each substance and concentration (Table 1) in parallel in 6 test wells (minimum *n* = 6).

**Table 1:** Concentrations of the enzymes, surfactant, and chelator with respective buffer solutions used for the tests

Test substances	Buffer solution used	Enzyme test concentrations (µg/ml)		Mean enzyme activity (U/mg)
		Tested in cytotoxicity assay mean and error bar (max. reduction in %)	Used for biofilm treatment	
Enzymes				
α-amylase	DPBS		6000	361 [227–445]
Benzonase^®^	DPBS added with Mg^2+^		0.07	136
Dextranase	DPBS		200	–
DispersinB^®^	DPBS		250	37
Lysozyme	DPBS		2500, 5000	2590 [940–4500]
Subtilisin A	DPBS		2	1307
Others				
CAPB	DPBS without Mg^2+^ and Ca^2+^		10	–
EDTA	PBS without Mg^2+^ and Ca^2+^		292	–

The mean and maximum reduction value in comparison to the control of the cytotoxicity test (reduction in %) and concentration of test substances that were used to treat the biofilms are presented. The number of samples was 12 or 18 for each test substance. Additionally, the measured activity of the enzyme is listed.

*DPBS* Dulbecco’s phosphate-buffered saline, *CAPB* cocamidopropyl betain, *EDTA* ethylene diamine tetraacetic acid

### Test specimens

Sandblasted, roughened titanium discs (Ø 5 mm, arithmetical mean deviation *R*_a_ = 830 nm, average surface roughness *R*_z_ = 3690 nm; Sirona, Bensheim, Germany) were used for the study.

### Biofilm culture

Biofilm was cultured as described previously [17] and thus is only briefly described here. Using curettes, the inoculum was harvested from subgingival plaque in deep pockets of the same periodontally diseased volunteer (approved by the Ethics Committee of University Medicine Greifswald, Registration number: BB 049/16) for each of the experimental runs, and was pooled and stored for a maximum of one week at 4 °C in culture medium (Dulbecco′s modified Eagle medium DMEM; Invitrogen GmbH, Karlsruhe, Germany) with 10% foetal calf serum (FCS; PAA Laboratories, Pasching, Austria), adapted from an established plaque biofilm model [18]. The stored plaque was incubated 24 h at 37 °C before biofilm culturing was initiated.

The titanium discs were placed into 96-well microtitre plates (Techno Plastic Products AG, Trasadingen, Switzerland), covered with 100 μl of subgingival human plaque suspension and cultured for 7 days at a constant temperature of 37 °C in an incubator (Serie BC; Binder GmbH, Tuttlingen, Germany) with 5% CO_2_ (approx. 5 kPa) and > 90% relative humidity (via water bowl inside the incubator) under aerobic conditions. The culture medium was replaced every 24 h.

The biofilm thickness of one test run was determined by confocal laser scanning microscopy (LSM 510 Exciter, Carl-Zeiss, Germany) after staining with the fluorescent dye acridine orange (4 µg/ml) (Carl Roth, Karlsruhe, Germany) for visualisation.

The microbial composition of the cultured biofilm was analysed once externally for typical oral species (micro-IDent plus, Hain Lifescience, Nehren, Germany).

The average biofilm thickness was measured on 3 discs bearing biofilm cultures using a confocal laser scanning microscope (Zeiss CLSM510 Exciter; Carl Zeiss Jena, GmbH, Jena, Germany).

### Biofilm destabilisation

The culture medium was replaced by 80 µl of the test substances in each microplate well; incubation was performed for 15 min at 36 °C.

After incubation, the specimens were washed once and refilled with 150 µl DPBS, then shaken for 15 min at 400 rpm on a microplate shaker to generate shear stress. Based on the mean grey values of the digital images (ImageJ, v1.50, US National Institutes of Health, Bethesda, MD, USA), the biofilms were analysed on fluorescence microscopy images after staining with the fluorescent dye acridine orange (4 µg/ml) (Carl Roth, Karlsruhe, Germany). Therefore, the microscope Olympus BX 60 (Olympus, Hamburg, Germany) with a mercury-vapor lamp, a 2× lens, and an adapter to connect a Canon EOS 450D camera (Canon Inc., Tokyo, Japan) was used to obtain an image of the total disc area. The setting of the camera, white balance, and exposure time at 2.5 s remained constant to guarantee comparable images.

To determine the reduction of biofilm mass, the test runs were repeated at least four times with six test wells per microplate of each substance and concentration, where up to five substances were tested in parallel. Within one test run, the test substances were grouped in a maximum of two separate microplates with preincubated biofilms on titanium discs.

The values after treatment with the test substance were compared by detailed statistical analyses with the values of the negative control, which was treated only with the carrier solution DPBS.

If the mean value of the negative control was lower than 80,000,000, which is equivalent to a density value of 113, the test run was excluded from statistical analyses to maintain a stable range of biofilm thickness and therefore comparability between results of different test runs. When the positive control (CAPB 2%, and 5%) failed, the test run was also not used for analysis. This led to varying numbers of samples at the end of the statistical analyses.

### Statistical analysis

The biofilm reduction values are based on the mean grey values of the histogram of the specimen images of a test group. Therefore, the grey values were calculated using the Histogram Integrated Density with program ImageJ. The greyscale value span of the digital 8-bit images ranges from 0 (no fluorescence signal) to 255 (highest possible fluorescence signal). Data on biofilm destabilising effects were presented as means and standard deviations (SDs). Mixed-effects linear regressions were used to estimate effects of different treatment methods (treatment as a fixed effect; with DPBS as the reference) on biofilm reduction values, including a random intercept for different runs. Robust standard errors were estimated. Due to the explorative character of the present pilot trial, p-values were not corrected for multiple testing.

The four test runs were excluded if the controls were out of order. *P* values < 0.05 were considered statistically significant. Analyses were conducted with Stata/SE (StataCorp. LP, Stata Statistical Software: Release 14.2 College Station, TX, 2015).

## Results

A summary of the different concentrations used for the cytotoxicity tests and those used for biofilm destabilisation (below 50% cell viability reduction), as well as the mean enzyme activities, are listed in Table 1. An application concentration within acceptable tissue-tolerable cytotoxicity was found for all test substances (Table 1).

### Biofilm destabilisation effect was shown by the enzyme lysozyme

The biofilms were exposed to test substances for 15 min. After exposure and shear stress procedure, only lysozyme (2500 μg/ml) showed a statistically significant biofilm reduction (13.7 lower, correspond 10% reduction), as indicated by fluorescence signals (greyscale value of the histogram). The other test solutions showed no significantly reduced mean greyscale values in comparison to the control (Table 2).

**Table 2:** Measurements of the effects of test substances on biofilm

Test substances	*N*	Mean ± SD	Mean difference to control	Mixed-effects linear regression^a^	*p *value
				B (95% CI)	
Enzyme**s**					
α-amylase	36	137.1 ± 12.3	2.0	− 4.2 (− 8.3; − 0.1)	0.045
Benzonase®	42	136.2 ± 9.9	1.1	− 2.5 (− 6.0; 1.0)	0.160
Dextranase	30	130.8 ± 17.4	− 4.3	− 7.1 (− 15.1; 1.0)	0.090
DispersinB^®^	23	138.8 ± 16.9	3.7	4.6 (− 0.7; 10.0)	0.090
Lysozyme 2500	27	121.4 ± 14.3	− 13.7	− 3.9 (− 6.9; − 1.0)	0.009
Lysozyme 5000	36	134.2 ± 21.6	− 0.9	− 0.03 (− 5.2; 5.2)	0.990
Subtilisin A	30	131.5 ± 16.9	− 3.6	− 1.0 (− 5.3; 3.3)	0.650
Others					
CAPB	24	132.5 ± 16.1	− 2.6	1.4 (− 0.7; 3.5)	0.200
EDTA	30	141.2 ± 11.3	6.1	0.6 (− 2.1; 3.4)	0.650
Control					
DPBS	58	135.1 ± 13.3		0 (ref.)	
Test control (positive control)					
CAPB 2%/5%	63	85.4 ± 31.1	− 49.7	(no statistic)	

Values are based on the digital image analysis of pixels that show fluorescence signals and were compared to the control with DPBS. Number of cumulative samples (*N*) for each run, the mean and standard deviation (SD), as well as values of the regression model, confidence interval, and resulting *p *values are shown

*N* number of samples, *SD* standard deviation, B, regression coefficient; CI, confidence interval; CAPB, cocamidopropyl betain; EDTA, Ethylenediaminetetraacetic acid; DPBS, Dulbecco's Phosphate-Buffered Saline

^a^Mixed-effects linear regression with robust standard errors and a random effect for test runs

### Biofilm properties

**Fig. 2:**
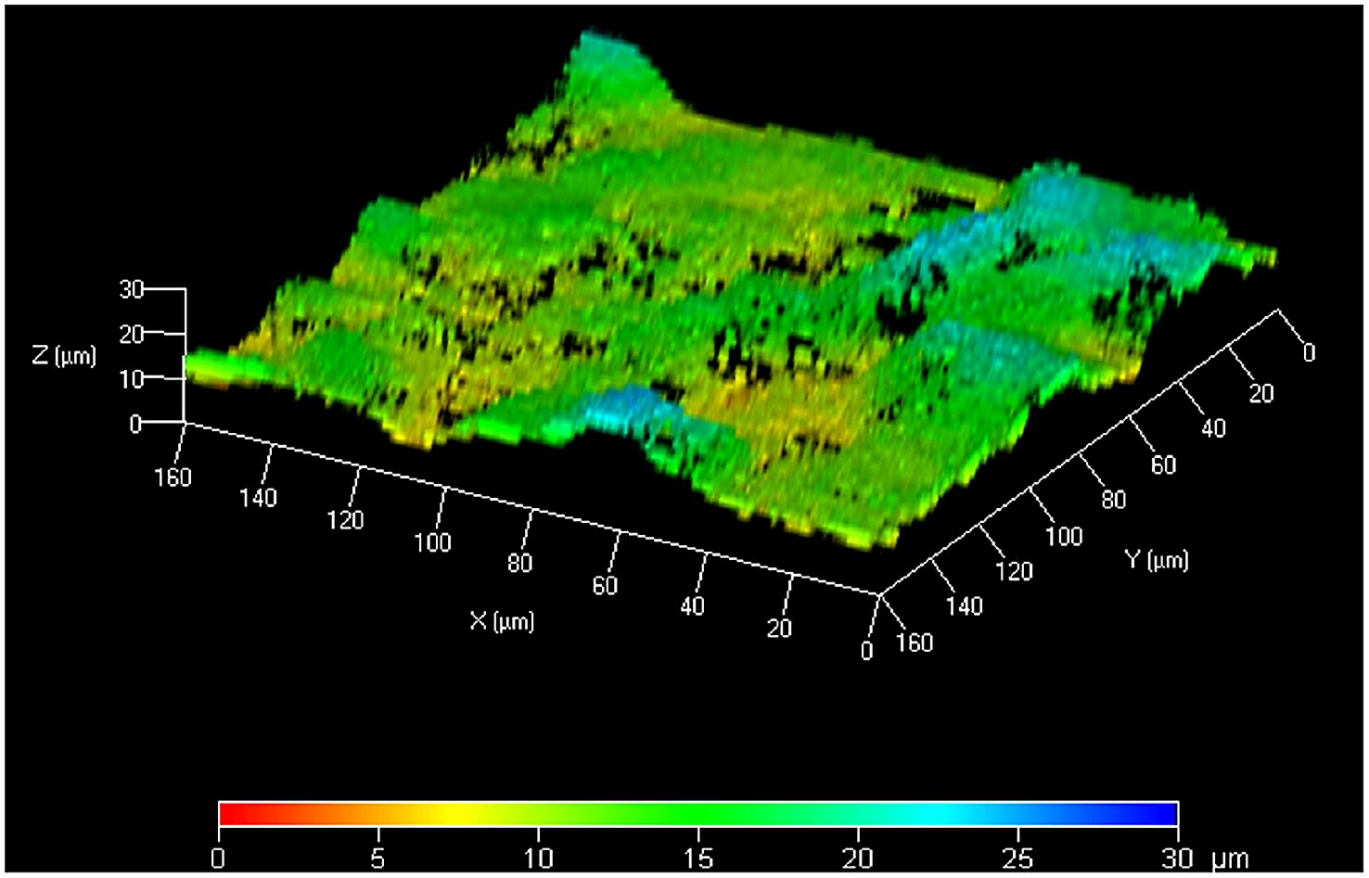
3D height image of a plaque biofilm. An x–y–z-surface colour plot of a biofilm (axis scale in µm), cultured on rough titanium discs, stained with acridine orange (Microscope: LSM 510 Exciter, Carl-Zeiss, Jena, Germany) is shown

The mean biofilm thickness was 25.4 ± 5.3 µm (Fig. 2).

The species *Porphyromonas gingivalis*, *Tannerella forsythia*, *Peptostreptococcus micros*, *Fusobacterium nucleatum*, *Campylobacter rectus* and *Eiknella corrodens* were detected by commercial molecular testing of the biofilm.

## Discussion

Residues of microbial biofilms can interfere with successful re-osseointegration supported by guided surgical procedures after implant cleaning with conventional mechanical methods because the complex, microrough surface and the screw shape of implants impedes effective surface treatment. This study investigated the biofilm destabilising effect of different enzymes, a surfactant, and a chelating agent to find a method which could support mechanical cleaning procedures during flap surgery. This early stage of investigations does not claim to optimally mimic clinical conditions, but this will be necessary in further experiments to evaluate defined treatment regimes. For that reason, 10% biofilm removal, achieved by shear stress in combination with lysozyme (2500 µg/ml), is alone too little to be clinically relevant. However, the test substances, especially the enzymes, will presumably not be able to remove microbial biofilms alone at cell-tolerable concentrations and can only work as adjuncts to support a multistage procedure, e.g., combined with mechanical cleansing. Especially for dental implant cleansing, the microrough surface hampers complete biofilm removal. Destabilising the biofilm matrix could improve biofilm removal by other treatment methods and therefore lead to increased healing success and long-term health after surgical biofilm removal. Hence, this study was planned so that biofilm removal was not directly tested after incubation of test substances, but was tested after subsequent mechanical shear stress, to detect the destabilising effect (and not the direct removal ability) of test substances. The antimicrobial effect or inhibition of biofilm formation was not tested so that a potential resistance of microorganisms against the tested substances or interactions and pellicle formation within the oral cavity did not play a role here.

Biofilms were cultured for 7 days to achieve a mature, stable mixed-species biofilm with relevant oral microorganisms, which was confirmed by external analysis, despite aerobic culture (with 5% CO_2_) using an established subgingival biofilm model [18].

In further studies, a more detailed analysis of microbial composition and chemical analysis is planned to promote the understanding of chemical or enzyme action. The conditions of the in-vitro biofilm culture in this study cannot imitate the conditions of the in-situ environment, even if the microbial composition of the subgingival biofilm of periodontitis display similarities with the microbial composition of peri-implantitis [19].

The biofilm structure of the tested specimen was analysed using fluorescent dyes (data not shown); the biofilm contained polysaccharides, proteins, and DNA, thereby confirming that the application spectrum of the tested substances indeed attacked the biofilm matrix. The different glycosidases act on α-1,4- (amylase), α-1,6- (dextranase), β-1,4- (lysozyme, between *N*-acetylmuramic acid and *N*-acetyl-d-glucosamine), and *N*-acetyl-(DispersinB^®^) glycoside bonds, which exist in oral-biofilm exopolysaccharides [20, 21] or on cell surfaces [22]. Likewise, proteins and extracellular nucleic acids are structural elements of biofilms[21], which may be targeted for a protease (subtilisin A) or a nuclease (Benzonase^®^). Because biofilm matrix contains multivalent cations [8], which increase matrix stability, a chelator (EDTA) could help to remove them. Structures containing lipids are other targets which can be destabilised by a surfactant (CAPB). The incubation time of 15 min was chosen as the application time for therapeutic chairside use (this will be reduced in further studies).

With regard to future chairside use, the a priori decision was made to use a tissue-tolerable concentration of the selected test substances. Three to six different concentrations were tested to determine which promised acceptable cytotoxicity, as determined by the MTT assay on human cells. As expected, the MTT assay for attached cells failed for CAPB and EDTA, because these substances detached cells from the surface and thus led to seemingly reduced cell vitality. To obtain a more realistic impression of the cytotoxicity of CAPB and EDTA, detached cells in suspension were stained with trypan blue and observed in a cell chamber using a microscope. The result (data not shown) confirmed detachment of cells caused by EDTA and CAPB, whereas CAPB destroys the cell membranes, and EDTA was tolerable for cells up to 0.01 M solution. The incubation time of 15 min for the cytotoxicity test corresponded to the selected biofilm destabilisation time.

In the following paragraphs, the results for the tested substances are discussed in detail. Comparisons of the results for treatments with other studies are summarised in Table 3.

**Table 3:** Comparison of the enzyme results with other studies

Enzyme	Study	Biofilm reduction/incubation time and temperature	Concentration/enzyme activity	Origin of enzyme used	Used test organisms/biofilm model
Amylase	Craigen et al. [25]	90%/10 min, 37 °C	not specified	*Aspergillus oryzae*	*S. aureus*/18 h, 37 °C, on polystyrene
		Not significant/10 min, 37 °C	not specified	*Bacillus subtilis*	
		ca. 85%/3 h, 37 °C			
	This study	No significant reduction / 15 min, 36 °C	227–445 U/mg	*Bacillus subtilis*	Plaque biofilm/7 days, 37 °C, on titanium disks
Benzonase^®^	No specific reference for Benzonase available. Microbial biofilm destabilising effects by nucleases has been described elsewhere [32]				
Dextranase	Tsuchiya [38]	3.5%/20 min, 37 °C	1 kU/ml	*Paecilomyces lilacinum*	Co-culture of *S. mutans, A. viscosus, and F. nucleatum*/3 days, 37 °C, on hydroxyapatite disks
	This study	3.2%/15 min, 36 °C	0 U/mg	*Penicillium *sp.	Plaque biofilm/7 days, 37 °C, on titanium disks
DisersinB^**®**^	Chaignon et al. [31]	80% (only effective on PNAG producing strains)/2 h, 37 °C	970 U/mg	*Aggregatibacter actinomycetemcomitans*	*S. epidermidis and other (Staphylococcus *sp*.)*/24 h, 37 °C, on polystyrene
	This study	No effect	37 U/mg	*Aggregatibacter actinomycetemcomitans*	Plaque biofilm /7 days, 37 °C, on titanium disks
Lysozyme	Chen and Stewart [34]	40% (protein content)/60 min, 25 °C	50,000 U/mg (not measured)/500 µg/ml	from hen’s egg protein	co-culture of *P. aeruginosa* and *K. pneumoniae*/24 h, 25 °C, on 316L stainless steel slides
	This study	10% (biomass) /15 min, 36 °C	940–4500 U/mg/2500 μg/ml	from hen’s egg protein	Plaque biofilm/7 days, 37 °C, on titanium disks
Subtilisin A	Lefebvre et al. [33]	40%, 80% / 24 h, 37 °C	0.01 U/ml	*Bacillus licheniformis*	*P. aeruginosa, S. aureus*/24 h, 37 °C, on glass slides
	This study	−/15 min, 36 °C	1307 U/mg (2.6 U/ml)/2 µg/ml	*Bacillus licheniformis*	Plaque biofilm/7 days, 37 °C, on titanium disks
CPAB	There is no specific reference for cocamidopropyl betaine as the sole test substance to destabilize the microbial biofilm				
EDTA	Cavaliere et al. [37]	43%/1 h, 37 °C	25 mM	*–*	*Hemophilus influenzae*/24 h, 37 °C, on polystyrene
	Lefebvre et al. [33]	99.7%, 91.1%/24 h, 37 °C	20 mM	*–*	*P. aeruginosa, S. aureus*/24 h, 37 °C, on glass slides
	This study	–/15 min, 36 °C	1 mM	*–*	Plaque biofilm/7 days, 37 °C, on titanium disks

Important test parameters such as incubation time of the test substances, enzyme concentration and/or enzyme activity, the origin of the enzyme used, and biofilm model are presented to support the classification of the results in the context regarding the biofilm destabilizing effect by enzymes

The present results showed no significant biofilm destabilising effect of any of the tested substances, except lysozyme at 2500 μg/ml (Table 2). Interestingly, a higher concentration of lysozyme did not yield better results; the lower lysozyme concentration of 2500 µg/ml achieved a greater biofilm destabilising effect of 10.1% (value −13.7) than did 5000 µg/ml of 0.7% (value −0.9) (Table 2). This discrepancy could be caused by protein aggregation of high lysozyme concentrations [23] and should be avoided in further investigations. The wide range of enzyme activity (Table 1) cannot be identified as the sole cause of this effect based on the data from the individual experiments. The decreasing effectiveness to destabilise the biofilm by increased enzyme concentration was not recognisable for the other enzymes. Many studies have examined lysozyme in terms of avoiding biofilm development, but rarely to reduce or to destroy biofilm matrix. Besides the enzymatic activity of lysozyme (muramidase), lysozyme could also be helpful as an antimicrobial agent, because it disrupts bacterial membranes, thus controlling the infection [24], and is, therefore, a promising enzyme for treating biofilms.

Alpha-amylase can remove biofilms very efficaciously. However, its activity and specificity depend on the origin [25, 26]. Craigen et al. reported comparable results for an incubation time of 10 min[25]; however, a significant reduction was only found after 3 h. Therefore, the alpha amylase seems to be less suitable for chairside usage.

Dextranase only showed a biofilm destabilising effect after some test runs (data not shown). This may indicate instable dextranase activity in the present study. Apart from that, the present results of the activity and cytotoxicity tests were inconsistent. The mean activity of dextranase was 0 U/mg (no activity), but it did show a cytotoxic effect. Hence, the test concentration used had to be active. It is possible that the solution medium DPBS was unsuited to the activity test (Table 4).

**Table 4:** Remarks on the decision of the buffer solution used for enzyme tests

Dextranase	Dextranase in sodium–potassium-phosphate buffer showed an activity of 18 U/mg but was too cytotoxic and therefore not used for the main tests. A more suitable buffer solution, than DPBS, should be chosen in future tests to enhance the potential of dextranase to destabilise cohesiveness of the biofilm matrix
DispersinB^®^	Investigations about different carrier media showed that DPBS reduced the enzymatic activity of DispersinB^®^ compared to the recommended citrate buffer (pH 4.6), which, however, is not suitable for treating human cells
Benzonase^®^	The Mg-enriched DPBS used reduced the activity approximately fivefold, compared to the recommended TRIS–HCl (pH 8.0) buffer, which showed high cytotoxicity in pre-tests of this study, and may not be suitable for therapeutic chairside use

DispersinB® is a patented, widely studied enzyme to degrade biofilm matrix that contains poly-*N*-acetyl-glucosamine (PNAG) structures of streptococci and other bacteria or fungi [27–30]. The enzymatic activity with 37 U/mg, as used in the present study, is very low compared to other studies with 970 to 10^3^ U/mg protein [28, 31], seemingly caused by the buffer solution used (Table 4). This could be the reason why no biofilm destabilisation was observed in our test setup. Another reason could be a lower amount of PNAG in our plaque biofilms. DispersinB^®^ concentrations above 250 µg/ml in DPBS showed cytotoxic effects; thus, a higher activity could be associated with higher cytotoxicity.

Benzonase® is an often-used endonuclease to prepare harvested biofilm matrix for further protein or polysaccharide analyses. A destabilising effect of nucleases on microbial biofilm structure has been described elsewhere [32]. However, it did not exhibit any antibiofilm effect in the present study. The activity test resulted in 136 U/mg, which was approximately seven times lower than the expected 1000 U/mg of the original manufacturer’s solution, as calculated using data provided by the manufacturer that could be influenced by the used buffer solution (Table 4). Insufficient enzyme activity or a low impact of extracellular DNA in biofilm matrix could be the reasons for the lack of effect.

Subtilisin A is a widely investigated commercial proteinase for removing biofilm mass. The concentration of 2 µg/ml enzyme resulted in an enzyme activity of 1307 U/mg or 2.6 U/ml without significant biofilm reduction. The study by Lefebvre et al. [33] demonstrated that a biofilm composed of different species can lead to different efficacy of proteases within one enzyme subgroup, which makes it difficult to find a generally effective protease to destabilise biofilms in clinical practise.

Surfactants can very effectively remove biofilms [34]. CPAB, derived from coconut oil and dimethylaminopropylamine, was used for this study because it is widespread in cosmetic products (especially in washing-up liquids) with an acceptably low irritant potential [35], as well as in wound cleaning solutions [36], which suggests high tissue tolerability. However, based on the present cytotoxicity test, it was possible to accept only a very low concentration of 10 µg/ml (0.001%), which was not active enough to significantly remove biofilm mass in the present tests. However, CAPB was used as the positive control of each test run with 2 and 5% solutions, resulting in a mean reduction of 37.8% (data not shown). A detergent with such a high concentration, though capable of destabilising the biofilm matrix, is not tissue compatible and, in addition, annoying foaming may occur.

The chelator EDTA is able to support biofilm removal in in-vitro biofilms [33, 37]. The EDTA concentration selected here was very low (1 mM) to avoid human cell detachment. The results showed that 1 mM ETDA was insufficient to destabilise in-vitro dental biofilms, thus making it unsuitable as a stand-alone application. It is possible that 1 mM ETDA has an additive effect in combination with enzymes that are not dependant on divalent cations.

Combining enzymes/chemicals may be a further option. Despite the low or absent effect of the tested enzymes, we assume beneficial effects of a combined use of different enzymes and/or the chelator/EDTA, even at low concentrations, to support mechanical cleaning. When different substances are combined for biofilm treatment, they might support each other by acting on different targets, which has already been shown for dextranase and mutanase [38, 39], or by improving penetration into deeper biofilm layers [40]. These results underpin the importance of simultaneously attacking different chemical targets in multispecies biofilms. The degrading effect of extracellular polysaccharides seems to be the main target, but it depends on the microbial strains involved; proteinases alone or combined with other substances can also be more effective in destroying biofilm cohesiveness than glycosidases [41, 42]. However, in practice, the plaque biofilm composition on the implant surface in any given patient will differ from that in another patient. This poses an additional challenge to find the right enzyme(s) or enzyme cocktail to achieve reliable treatment results.

## Conclusion

Periimplantitis therapy is a major challenge for the dentist. Treatments with enzymes could be a promising option to improve the mechanical cleaning process during flap surgery. Here, the only lysozyme showed significant biofilm destabilisation. Dextranase or Subtilisin A showed only a tendency to support the destabilisation of the biofilm within the cell-compatible application concentration. It is probable that lysozymes or other enzymes can also be used in other wounds, such as during debridement of open ulcerations or to support disinfection processes. Possibly, enzymes could also be used to clean contaminated implants in other parts of the body to reduce chronic inflammation.
